# Cool habitats support darker and bigger butterflies in Australian tropical forests

**DOI:** 10.1002/ece3.2464

**Published:** 2016-10-14

**Authors:** Shuang Xing, Timothy C. Bonebrake, Chin Cheung Tang, Evan J. Pickett, Wenda Cheng, Sasha E. Greenspan, Stephen E. Williams, Brett R. Scheffers

**Affiliations:** ^1^ School of Biological SciencesThe University of Hong KongHong KongChina; ^2^ School of Science and TechnologyThe Open University of Hong KongHong KongChina; ^3^ College of Marine and Environmental ScienceJames Cook UniversityTownsvilleQLDAustralia; ^4^ Department of Wildlife Ecology and ConservationUniversity of FloridaGainesvilleFL 32611USA

**Keywords:** climate change, morphology, trait, tropical forest

## Abstract

Morphology mediates the relationship between an organism's body temperature and its environment. Dark organisms, for example, tend to absorb heat more quickly than lighter individuals, which could influence their responses to temperature. Therefore, temperature‐related traits such as morphology may affect patterns of species abundance, richness, and community assembly across a broad range of spatial scales. In this study, we examined variation in color lightness and body size within butterfly communities across hot and cool habitats in the tropical woodland–rainforest ecosystems of northeast Queensland, Australia. Using thermal imaging, we documented the absorption of solar radiation relative to color lightness and wingspan and then built a phylogenetic tree based on available sequences to analyze the effects of habitat on these traits within a phylogenetic framework. In general, darker and larger individuals were more prevalent in cool, closed‐canopy rainforests than in immediately adjacent and hotter open woodlands. In addition, darker and larger butterflies preferred to be active in the shade and during crepuscular hours, while lighter and smaller butterflies were more active in the sun and midday hours—a pattern that held after correcting for phylogeny. Our ex situ experiment supported field observations that dark and large butterflies heated up faster than light and small butterflies under standardized environmental conditions. Our results show a thermal consequence of butterfly morphology across habitats and how environmental factors at a microhabitat scale may affect the distribution of species based on these traits. Furthermore, this study highlights how butterfly species might differentially respond to warming based on ecophysiological traits and how thermal refuges might emerge at microclimatic and habitat scales.

## 1 Introduction

Life‐history and functional traits related to morphology and physiology directly influence species dispersal and distributions (Jiguet, Gadot, Julliard, Newson, & Couvet, 2007; Musolin, 2007; Pacifici et al., 2015; Pöyry, Luoto, Heikkinen, Kuussaari, & Saarinen, 2009). Morphological traits with thermal consequences, such as color lightness or body size, interact directly with the environment and affect ectotherm body temperatures. The combination of exposure (morphology × environment) and sensitivity (morphology × physiology) is a primary determinant of how species will respond to climatic changes (Clusella‐Trullas, van Wyk, & Spotila, 2007; Kingsolver & Huey, 1998; Partridge & French, 1996; Walters & Hassall, 2006). Species must therefore balance thermal environments across space and time and manage their environmental niche given their traits and thermoregulatory abilities (Angilletta, 2009; Angilletta, Niewiarowski, & Navas, 2002; Magnuson, Crowder, & Medvick, 1979; Tracy & Christian, 1986).

The selection of temperature sensitive traits may drive divergence across thermal gradients such as elevation and latitude. For example, darker ectotherms absorb solar radiation more quickly and therefore tend to be more active and abundant at high elevations and in cooler poleward latitudes (Alho et al., 2010; Clusella‐Trullas, Terblanche, Blackburn, & Chown, 2008; Clusella‐Trullas, Wyk, & Spotila, 2009; Ellers & Boggs, 2004; Guppy, 1986; Roland, 2006). In a key continental‐scale study, Zeuss, Brandl, Brändle, Rahbek, and Brunzel (2014) documented darker butterfly and dragonfly assemblages in cold environments across elevation and latitude gradients in Europe. In addition to color, large body size also provides physiological benefits for living in cold environments due to low convection rates and higher heat capacities (Porter & Gates, 1969). As a consequence, body size of ectotherms is often negatively correlated with environmental temperature, resulting in body size clines across thermal gradients (Atkinson & Sibly, 1997; Kingsolver & Huey, 2008; Moreno Azócar et al., 2015; Partridge & French, 1996). Because many morphological and physiological traits are linked to climate and the surrounding environment, recent evidence has emerged showing that climate change is triggering increased color lightness and decreased body size for numerous ectotherm species (Angilletta, Niewiarowski, Dunham, Leaché, & Porter, 2004; Gardner, Peters, Kearney, Joseph, & Heinsohn, 2011; Zeuss et al., 2014; but see Connette, Crawford, & Peterman, 2015).

One possible mediator of climate change impacts on biological communities is microhabitats, which are generally decoupled from macroclimatic gradients and thus offer unique thermal regimes for organisms to persist in situ under climate change (Scheffers, Edwards, Diesmos, Williams, & Evans, 2014; Scheffers, Evans, Williams, & Edwards, 2014). Differences in microclimates within and among habitats, such as closed‐canopy forests and open habitats, can be comparable to or greater than gradients across altitude and latitude (Huey et al., 2009; Mark & Ashton, 1992; Scheffers et al., 2013). For small ectotherms that operate at the scale of microhabitats, microscale climate systems are especially important (Bonebrake, Boggs, Stamberger, Deutsch, & Ehrlich, 2014; Pincebourde & Casas, 2015; Potter, Arthur Woods, & Pincebourde, 2013). Another important mechanism in efficient thermoregulation is the adjustment of activity times during the day or throughout the year (Porter, Mitchell, Beckman, & DeWitt, 1973; Stevenson, 1985), which entails finding temperatures at both macro‐ and microscales that optimize performance (Clusella‐Trullas, Blackburn, & Chown, 2011; Grant, 1990; Kearney, Shine, & Porter, 2009).

In this study, we examined the role of temperature and butterfly morphology across tropical forest habitats in north Queensland, Australia, to further understand how thermal gradients at microclimate and habitat scales interact with traits of species assemblages. Butterflies are particularly diverse in morphology, especially in color and size (Beldade & Brakefield, 2002), and are sensitive to ambient temperature and solar radiation (Kingsolver, 1985; Ohsaki, 1986). They therefore serve as model organisms for testing the consequences of morphology in structuring community traits at micro‐ and macrohabitat scales and under climate change (Bonebrake et al., 2014; Kingsolver, 1985; Kingsolver & Buckley, 2015). In this study, we first examined species compositions across disparate environments (hot and cool) and linked species distributions in closed and open forest sites to color and body size morphology. We then experimentally examined how color lightness and body size of mounted specimens affect their body temperature change under controlled conditions. Finally, we assessed the influence of evolutionary history on these color traits by constructing a phylogeny and analyzing the effect of phylogenetic and species‐specific contributions to measured trait values. The results provide insights into how the environment interacts with morphology to structure communities across microclimatic and habitat scales as well as how that variation could have important implications for how biodiversity will respond to climate change.

## 2 Methods

### 2.1 Field surveys

We sampled butterflies in primary rainforests and open woodland habitats of the Australian wet tropical (AWT) bioregion in northeastern Queensland, Australia. Within this region, we conducted our sampling at two locations: Daintree Rainforest National Park (15°57ʹS, 145°24ʹE) and Shiptons Flat (15°42ʹS, 145°13ʹE), from 20 October to 1 Novembr 2014. We used five primary rainforest sites in the Daintree and two primary rainforest sites in Shiptons Flat. We also sampled in two open woodland sites in Shiptons Flat. We used hand nets and binoculars to survey active butterflies in crepuscular (7:00–10:00 a.m. and 3:00–6:00 p.m.) and midday hours (10:00 a.m.–3:00 p.m.) in each habitat (open woodland and primary rainforest). We surveyed along 0.5‐km transects for 30‐minute intervals, and we marked each captured butterfly to avoid double counting during each sampling. For those individuals not easily identified during sampling, we collected the specimen for identification in the laboratory. Additionally, we collected and mounted at least one specimen for each species for color lightness analysis.

We recorded the time of each capture and categorized the spot of capture as either sunny or shady. We used one Thermochron iButton data logger (model: DS1921) in each habitat to record the ambient temperature throughout the sampling period. Each temperature logger was suspended approximately 1.5 m above the ground and hung beneath a plastic funnel to avoid direct sunlight (Scheffers et al., 2013; Shoo, Storlie, Williams, & Williams, 2010).

### 2.2 Collection of morphology information

We photographed specimens using standardized settings (exposure time: 1/200 s, ISO speed: 100, aperture: F/16) and a flash from a fixed distance to record color. Basking postures for absorbing heat may vary by species, so we took photographs of both the dorsal and ventral sides of the wings for each specimen. The lightness value of each species was obtained using Adobe Photoshop CC 2014 software. Because studies have shown that the basal part of the wing and body are important for thermoregulation (Kingsolver, 1985; Wasserthal, 1975), we analyzed the color of the body, together with the one‐third wing area immediately adjacent to the body. We also analyzed the color of the body and the whole specimen (whole wing area plus body) to test the consistency of the lightness value across regions of the specimens. We used the quick selection tool in Photoshop to choose each region for color analysis and averaged the chosen region using the average blur filter. We used the mean of the value of the red, green, and blue channels as the final estimated lightness value (a value between 0 and 255—low values denote dark butterflies and high values indicate lighter individuals). For most of the species, we picked one best‐preserved specimen to represent the morphology. For species with multiple specimens, we took the average color lightness of all specimens, averaging male and female lightness values when specimens of both sexes were available. As wingspan is known to be an effective and convenient proxy for body size (Sekar, 2012), we collated wingspan (the distance between the tips of the forewings) data from the Butterflies of Australia field guide as an indicator of body size (Braby, 2000).

### 2.3 Thermal experiment

Using the best‐preserved collected mounted specimens for 26 species, we conducted an experiment with a FLIR thermal infrared camera (model: E6) over the course of two sunny days from 12 to 3 p.m. Our E6 model records 19,200 samples of temperature per photograph (160 × 120 pixels). This time period was chosen to ensure stable and strong solar radiation. We standardized our start temperatures by placing mounted specimens in full shade and exposed each specimen to full sun for 2 min. Using the thermal camera, we recorded the surface temperature of the thorax every 30 s. In this way, we collected starting ambient shade temperature of the thorax in addition to four sun‐exposed thorax temperatures.

### 2.4 Phylogenetic reconstruction

We obtained sequences for 37 of the 46 species recorded during sampling from the National Center for Biotechnology Information (http://www.ncbi.nlm.nih.gov/). We additionally selected another 16 species of Lepidoptera for phylogenetic reconstruction. Of these 53 species, we used 48 butterfly species as in‐group species and five moth species (*Mathoris loceusalis*,* Morova subfasciata*,* Rhodoneura terminalis*,* Striglina cinnamomea*, and *Thyris fenestrella*) as out‐group species. We used sequences from mitochondrial gene regions (COI, COII, 16s, and NADH5) and combined nuclear genes (CAD, EF1α, GAPDH, IDH, and *wingless*). We aligned the sequences using MAFFT multiple sequence alignment software version 7.215 (Katoh & Standley, 2013). Then, we concatenated and edited sequences manually in Geneious v.4.8.5 (Kearse et al., 2012). The aligned concatenated matrix contained 7,270 base pairs.

We performed Bayesian inference phylogenetic reconstruction with MrBayes v.3.1.2. (Huelsenbeck & Ronquist, 2001; Ronquist & Huelsenbeck, 2003). We used two independent MCMC chains, each with 24,000,000 generations, and sampled every 2,000th generation. We kept all other priors as default except cold Markov chain with a temperature parameter of 0.16, and we adjusted the mean branch length prior to 0.01 (brlenspr = unconstrained:exponential(100.0)) to reduce the likelihood of stochastic entrapment in local tree length optima (Brown, Hedtke, Lemmon, & Lemmon, 2010; Marshall, 2010). The resulting standard deviation of split frequencies was <0.01. The parameters sampled during the MCMC were imported into Tracer v.1.5 showing adequate effective sample size (>200, Rambaut & Drummond, 2007). We excluded 4,000 initial samples (around 33%) as burn‐in of each MCMC run from the summary analysis, and we calculated a 50% majority‐rule consensus tree from the post‐burn‐in trees. The posterior probabilities (PPs) of each node were summarized (Larget & Simon, 1999), and we used these to infer support for individual clades: nodes with PP values ≥.95 were regarded as well or strongly supported (Yang & Rannala, 1997).

### 2.5 Phylogenetic analysis

We extracted 1,000 trees randomly from the post‐burn‐in trees in one of the MCMC runs. We removed the 16 taxa which were added for phylogenetic reconstruction (*Cupido argiades*,* Papilio polytes*,* Euploea midamus*,* Hypocysta pseudirius*,* Mycalesis fuscum*,* Pantoporia sandaka*,* Ypthima confusa*,* Catochrysops panormus*,* T. fenestrella*,* M. subfasciata*,* R. terminalis*,* M. loceusalis*,* S. cinnamomea*,* Nacaduba angusta*,* Motasingha trimaculata*, and *Hasora chromus*) using Mesquite v.2.7.5 (Maddison & Maddison, 2011). For each sampled tree, we used Pagel's λ (Pagel, 1999) to calculate the phylogenetic signal of traits with the *phylosig* function in the R package *phytools* (Revell, 2012). We then rescaled the traits value by Pagel's λ using the *rescale* function in the R package *geiger* (Harmon, Weir, Brock, Glor, & Challenger, 2008) and used Lynch's comparative method (Lynch, 1991) to separate the total trait values to phylogenetic components and specific components for the 1,000 trees with function *compar.lynch* in R package *ape* (Paradis, Claude, & Strimmer, 2004). The phylogenetic components represent the ancestral contribution to the trait, while the specific components represent the species‐specific variance of the trait (Zeuss et al., 2014). In this way, 1,000 results for phylogenetic components and specific components for each species were generated.

### 2.6 Statistical analysis

#### 2.6.1 Community analysis

For all species for which we obtained abundance and environmental information, we used individual‐based rarefaction curves to compare the species richness of rainforest and woodland habitats. We also conducted a nonmetric multidimensional scaling (NMDS) to examine community‐level differences across habitats and sites, using each sampled location as a replicate. We excluded one rainforest site in the Daintree (Mt. Sorrow) from the community analysis due to the extreme low abundance of butterflies. We then conducted a PERMANOVA test using the *adnois* function in the R package *vegan* (Oksanen et al., 2013) to quantify the difference in community composition across habitats.

#### 2.6.2 Temperature gradient

We extracted the ambient temperature for each point in time at which a butterfly was observed or captured using data from the iButton data loggers. We then applied a multiple linear regression model to estimate the temperature gradient experienced by butterflies across habitat and time (crepuscular and midday hours) with the *predict* function in the R package *stats* (R Core Team, 2013).

#### 2.6.3 Thermal experiment

We applied linear mixed‐effects models to estimate the contribution of color lightness and body size to the observed increase in body temperature during the thermal experiment. As the thermal experiment was conducted over 2 days, we used day as a random factor and color lightness and wingspan as fixed factors. We used the *lme* function from the R package *nlme* (R Development Core Team, 2013). We performed a linear correlation of color lightness between whole wing with body, basal wing with body, and body only to test the consistency of color lightness across these body regions. We constructed two models with and without an interaction between color lightness and wing size and used Akaike information criterion (AIC) model selection to choose the model with the lowest AIC values for analyses (Akaike, 1974).

#### 2.6.4 Relationship between butterfly morphology and environmental factors

To relate habitat and assemblage trait characteristics, for each individual, we used the species‐specific morphological data as well as the environmental data (habitat, sun vs. shade) recorded at the time when the individual was observed. We applied a multiple linear regression to analyze how butterfly color lightness and body size was affected by habitat, time, and sunny/shady conditions. We also constructed multiple linear regression models with and without interactions between different environmental factors and applied AIC to choose the model with the lowest AIC value as the best‐fit model for morphology and environmental gradient analyses. We then used the *predict* function in the R package *stats* (R Development Core Team, 2013) to estimate butterfly color lightness and body size across the gradients generated by different factors based on the best selected models. We used the whole dataset (46 species with 408 individuals) for the analysis above. For the 1,000 phylogenetic components and specific components generated by the 1,000 phylogenetic trees for traits of each species (see the section 2.5, phylogenetic analysis), we separately ran the same selected multiple linear regression model for each component against environmental factors. We then used the *predict* function to estimate phylogenetic and specific components at each of the gradients based on the linear regression model results and averaged across the 1,000 estimates. Due to a lack of phylogenetic information for some rare species, we used a subset of the dataset (37 species with 363 individuals) for the phylogenetic analysis. All analyses were conducted in R v.3.2.2 (R Development Core Team, 2013).

Although our focus in this analysis was on individuals, we also used species as a unit to look into the presence–absence of different species traits across environmental gradients by applying the lowest AIC model for traits and environmental factors. Finally, in order to assess how variation in the abundance of common species across gradients might be affecting the observed patterns, we chose the top five most‐abundant species separately from our rainforest and woodland habitats and plotted the abundance of those species at each combination of light exposure, time of day, and habitat together with corresponding morphological data.

## 3 Results

### 3.1 Summary of butterfly assemblages

We recorded 408 individuals of 46 species through capture and binocular observation (Figs S1 and S2). Species richness was higher in rainforest (38 species) than in woodland (19 species; 11 species were found in both habitats; Fig. S3). The NMDS plot demonstrated some differentiation of the community composition between rainforest and woodland but no significant difference by the PERMANOVA test (*F* = 1.15, *p* = .17; Fig. S4).

The phylogenetic relationships of the species were summarized in Fig. S5. The phylogenetic relationship between different families in our study was similar to previous work (Heikkilä, Kaila, Mutanen, Peña, & Wahlberg, 2012; Regier et al., 2013). Although the relationship of the out‐group was ambiguous, the “true butterflies” were shown to be monophyletic (PP = .98). All deep nodes were strongly supported. The families Nymphalidae and Hesperiidae form a strong supporting clade sister to the clade containing the families Pieridae and Lycaenidae. Papilionidae is basal to the rest of the families.

### 3.2 Temperature gradients

Ambient temperature experienced by butterflies was significantly different between habitats and time of sampling (*R* ^2^ = .68, Fig. S6). The temperatures experienced in closed rainforests were approximately 2.3 ± 0.3°C (mean ± *SE*) lower than those in open woodlands, and temperatures experienced during crepuscular hours were approximately 3.4 ± 0.2°C (mean ± *SE*) lower than those experienced during midday hours. The coolest temperature observed in our study was approximately 25°C during crepuscular survey hours in rainforests, while the hottest temperature recorded was approximately 33°C during midday survey hours in open woodlands (Fig. S6).

### 3.3 Thermal experiment

Color lightness of three regions of the body and wing was highly correlated as indicated by linear regressions (*p *<* *.001, *R* ^2^ > .8). Given that the basal wing and body are considered the most important parts for butterfly thermoregulation (Kingsolver, 1985; Wasserthal, 1975), we focused on this region for subsequent analyses and note that here and elsewhere in the results and discussion “color lightness” refers to that of the basal part of the wing plus body. Our most parsimonious linear model suggests that color lightness negatively affected the rate of butterfly body warming under exposure to solar radiation, while wingspan positively affected warming (Table 1).

**Table 1:** Multiple linear regression models of color lightness and body size effects (mean ± *SE*) on experimental body temperature increase (lightness = color lightness of basal wing and body, size = wingspan)

Model factors	Lightness	Size	Lightness × size	AIC	∆AIC	LogLik
Lightness, size	−0.030 ± 0.009^a^	0.137 ± 0.028^a^		174.30	0	−82.15
Lightness, size, lightness × size	−0.070 ± 0.031^a^	0.094 ± 0.042^a^	0.001 ± 0.001	187.74	13.44	−87.87

AIC, Akaike information criterion; ∆AIC, Akaike differences; LogLik, the log‐likelihood estimate.

a *p < *.05.

### 3.4 Butterfly color lightness and body size across habitat gradients

**Figure 1:**
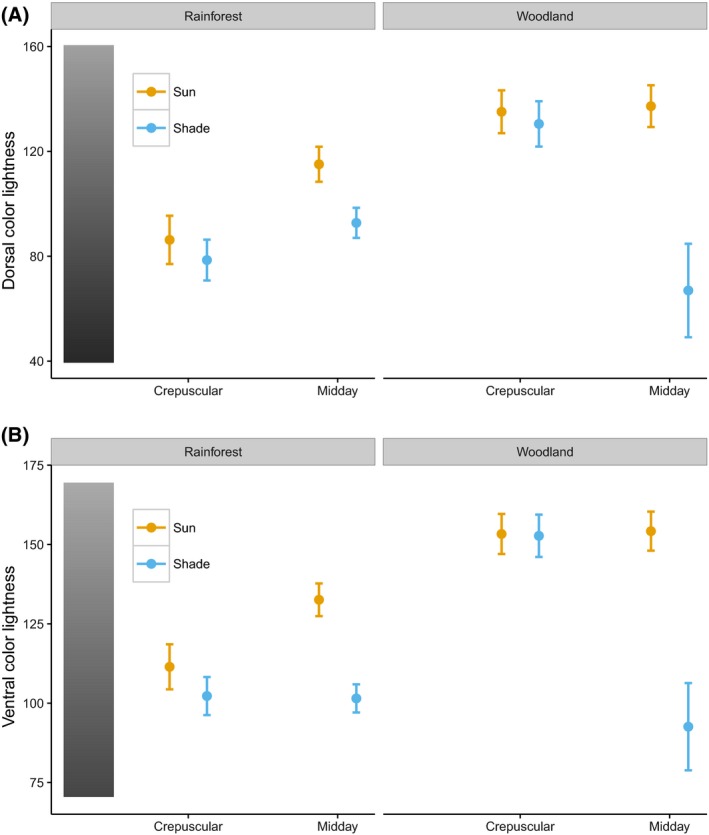
Predicted mean color lightness with standard error based on the best multiple linear regression model for dorsal (A) and ventral (B) sides of butterfly individuals across habitats and time (higher color lightness values indicate lighter colors) (*n = *326)

**Figure 2:**
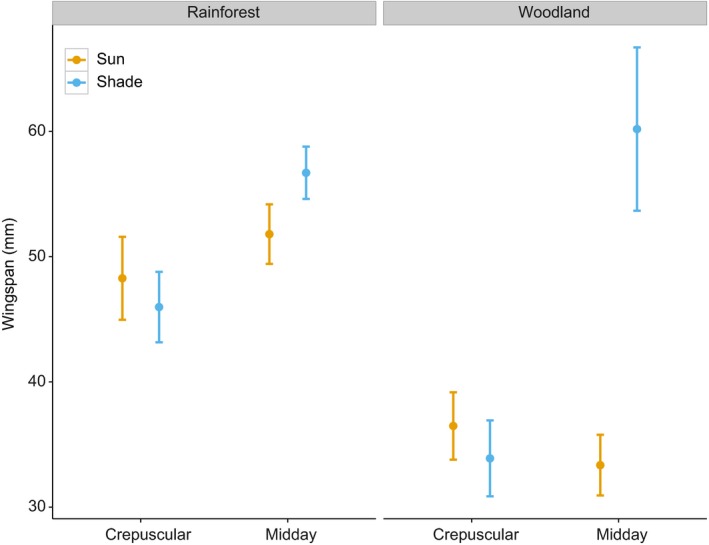
Predicted mean butterfly wingspan with standard error based on one of the best multiple linear regression models across habitats and time (*n* = 362)

Based on the model with the lowest AIC value (Table 2), we found that for both dorsal and ventral sides of the wings, color lightness of butterfly individuals predicted in closed rainforest was significantly darker than that found in open woodlands (Fig. 1) (Table 3). Body sizes of butterfly individuals in closed rainforests were also predicted to be larger relative to those in open woodlands (Fig. 2 and Table 3).

**Table 2:** Five best multiple linear regression models for environmental factors and butterfly color lightness

Factors	Number of factors	AIC	∆AIC	Weights	LogLik
Habitat, time, solar, time × solar, habitat × time, habitat × solar, habitat × time × solar	7	3,491	0	0.440	−1,736.5
Habitat, time, solar, time × solar, habitat × time	5	3,492.1	1.1	0.250	−1,739.063
Habitat, time, solar, time × solar, habitat × time, habitat × solar	6	3,492.6	1.6	0.202	−1,738.278
Habitat, time, solar, habitat × time	4	3,494.5	3.5	0.077	−1,741.239
Habitat, time, solar, habitat × time, habitat × solar	5	3,496.3	5.3	0.031	−1,741.153

AIC, Akaike information criterion; ∆AIC, Akaike differences; Weights, Akaike weights; LogLik, the log‐likelihood estimate.

**Table 3:** Statistical results of best multiple linear regression models for relationships between traits and environmental factors

Response trait	*df*	Parameter	Estimate	Std. error	*t* Value	*p* Value
Dorsal color lightness	318	Habitat (woodland/rainforest)	48.854	12.311	3.968	<.001
		Time (midday/crepuscular)	28.832	11.370	2.536	.01
		Solar (shade/sun)	−7.700	12.050	−0.639	.523
		Habitat × time	−26.704	16.115	−1.657	.099
		Habitat × solar	3.071	16.936	0.181	.856
		Time × solar	−14.654	14.926	−0.982	.327
		Habitat × time × solar	−50.996	27.305	−1.868	.063
Ventral color lightness	318	Habitat (woodland/rainforest)	41.865	9.500	4.407	<.001
		Time (midday/crepuscular)	21.129	8.774	2.408	.017
		Solar (shade/sun)	−9.202	9.298	−0.990	.3231
		Habitat × time	−20.245	12.435	−1.628	.105
		Habitat × solar	8.617	13.068	0.659	.510
		Time × solar	−21.883	11.517	−1.900	.058
		Habitat × time × solar	−39.153	21.069	−1.858	.06
Wingspan	354	Habitat (woodland/rainforest)	−11.796	4.267	−2.764	.006
		Time (midday/crepuscular)	3.526	4.079	0.864	.388
		Solar (shade/sun)	−2.297	4.345	−0.529	.597
		Habitat × time	−6.651	5.454	−1.220	.223
		Habitat × solar	−0.289	5.943	−0.049	.961
		Time × solar	7.196	5.377	1.338	.182
		Habitat × time × solar	22.225	9.681	2.296	.022

Within habitat, we observed a temporal and spatial effect on butterfly lightness. Specifically, in rainforests, individuals active during midday hours were lighter than those active during crepuscular hours (Fig. 1 and Table 3). In addition, individuals active in the sun were lighter than those in the shade, and this pattern became more pronounced during hotter midday conditions (Fig. 1). However, these patterns were not apparent in the woodland (Fig. 1). We found no significant effect of activity time and sun/shade level on wingspan (Fig. 2 and Table 3).

**Figure 3:**
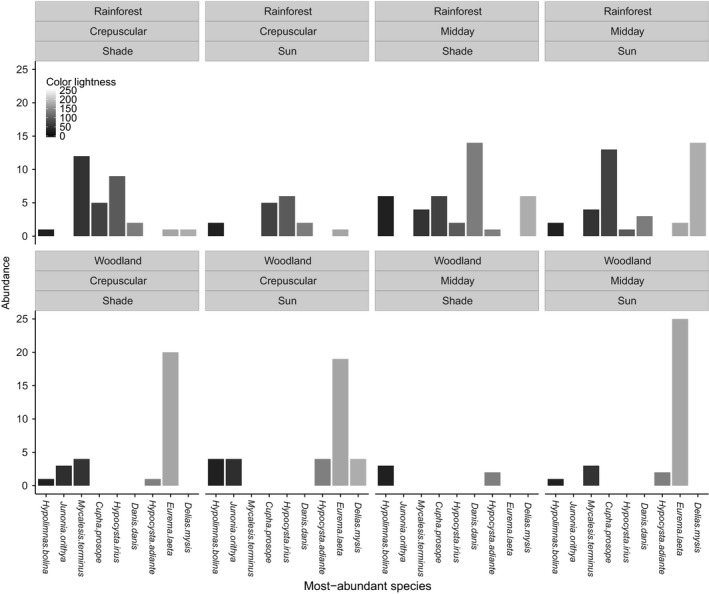
Abundance of the most‐abundant species within different combinations of light exposure, time of day, and habitat with their associated color lightness values presented

**Figure 4:**
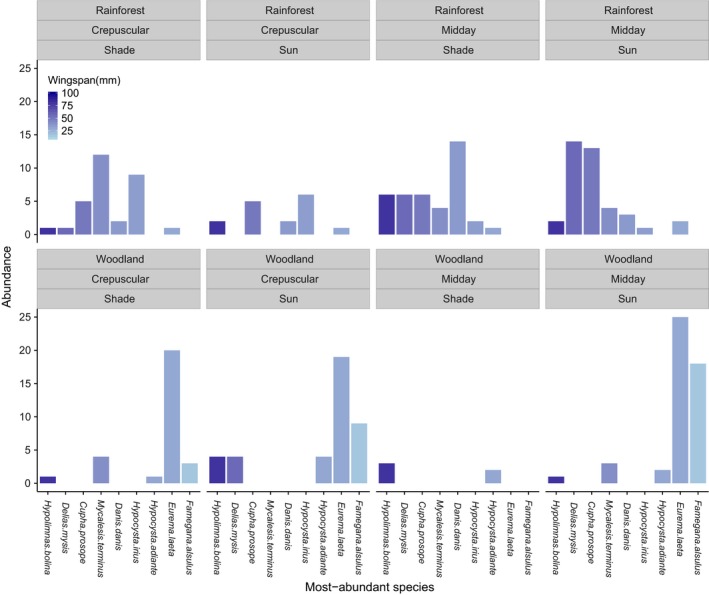
Abundance of the most‐abundant species within different combinations of light exposure, time of day, and habitat with their associated wingspan values presented

We found no significant difference in color lightness and size between microhabitats when data were analyzed at the species level (Figs S7 and S8). However, the abundance of the most‐abundant species for each habitat (five species from each habitat with only one abundant in both habitats: *Mycalesis terminus*), which account for near 70% of total abundance, varied by habitat and time of day (Figs 3 and 4). The most‐abundant species with darker colors are abundant under cool conditions such as in closed rainforests and during crepuscular hours (Fig. 3). Meanwhile, the most‐abundant species with relatively larger body sizes are abundant in closed rainforests and conversely those with relatively smaller body sizes are abundant in hot conditions such as open woodland and midday hours (Fig. 4).

**Figure 5:**
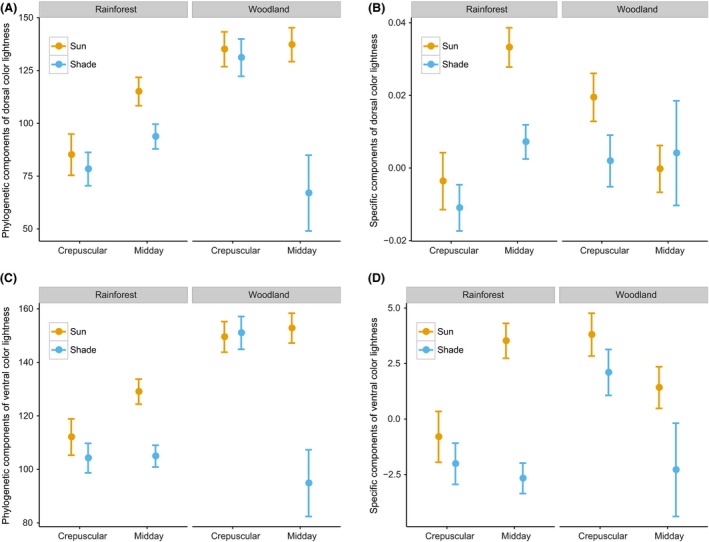
Predicted value based on the best multiple linear regression model of phylogenetic (A, C) and specific components (B, D) of mean color lightness with standard error for dorsal (A, B) and ventral (C, D) sides of butterfly individuals across habitats and time (*n = *319)

**Figure 6:**
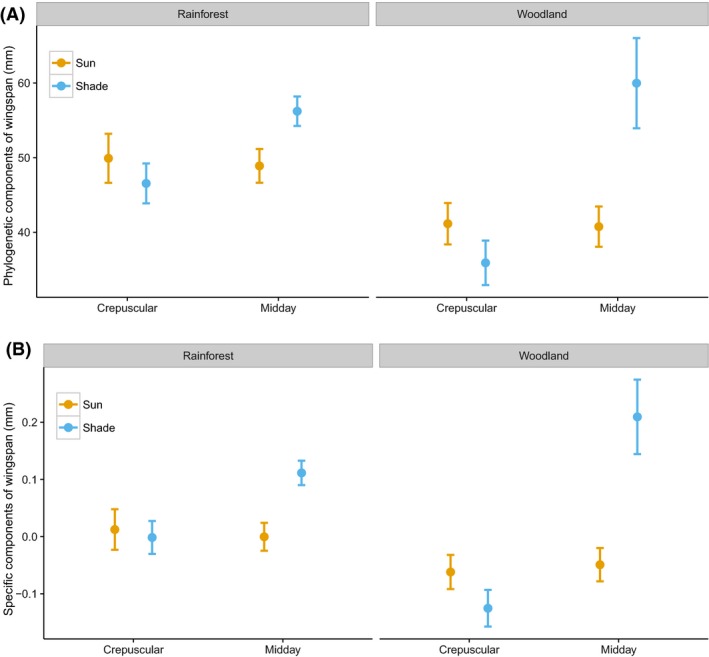
Predicted value based on one of the best multiple linear regression models of phylogenetic (A) and specific components (B) of mean wingspan with standard error across habitats and time (*n = *319)

Wingspan and color lightness were both highly phylogenetically correlated as lambda was greater than 0.9 for both traits (Fig. S9). Analyses of both phylogenetic components and specific components of color lightness and wingspan of butterfly individuals revealed that darker coloration and larger sizes were predicted in closed rainforest (Figs 5 and 6). Moreover, analyses of color lightness components showed that individuals active in the sun and midday were lighter than those active in the shade and crepuscular within rainforest. The pattern for specific components of color lightness across environment gradient was stronger for butterfly ventral side than for the dorsal side (Fig. 5).

## 4 Discussion

Our results suggest that darker and larger butterfly individuals tend to prefer cooler conditions both within and across habitats in tropical Australia. We observed consistent patterns of lightness and size across each level of exposure in space and time, spanning microhabitat (shade to sun), time of day (crepuscular to midday), and habitat (rainforest to open woodland). Although these patterns are highly influenced by phylogeny, the strong correlation between environmental factors and specific components shows that temperature gradients across habitats play an important role in structuring butterfly traits. The increased prevalence of dark and large individuals in cool conditions is supported by the results from our thermal experiment, which showed that dark bodies and larger sizes can accelerate heat gain, a finding consistent with other published research (Kingsolver, 1985; Porter & Gates, 1969; Wasserthal, 1975; Zeuss et al., 2014). Our findings support two prominent ecological hypotheses: that melanism scales with temperature (thermal melanism hypothesis) and that body size scales with temperature (Bergmann's rule) (Bergmann, 1847; Clusella‐Trullas et al., 2007; Gardner et al., 2011; Moreno Azócar et al., 2015; Partridge & French, 1996). Interestingly, we validated these two patterns at the interspecific level in the field—a finding which complements prior field research which has largely been focused on intraspecific variances at the population level, and macroecological research which has focused on large‐scaled distribution databases (Davis, Chi, Bradley, & Altizer, 2012; Ellers & Boggs, 2004; Moreno Azócar et al., 2015).

Temperature clearly differed between rainforest and open woodland sites even though these two habitats share a common ecotone. Our results suggest that trait variation can be shaped by these small‐scale thermal regimes (Baudier, Mudd, Erickson, & O'Donnell, 2015; Duffy, Coetzee, Janion‐Scheepers, & Chown, 2015)—a finding that is consistent with recent studies at the microhabitat (Baudier et al., 2015; Kaspari, Clay, Lucas, Yanoviak, & Kay, 2015) and ecosystem scale (e.g., temperate ecosystems, Moreno Azócar et al., 2015; Zeuss et al., 2014). Studies that focus solely on macroscale environmental gradients may be insufficient in resolution to detect ecotypic trait patterns of small‐bodied ectotherms, especially in complex environments such as tropical forests (Duffy et al., 2015).

Solar radiation also likely plays a significant role in shaping the pattern of butterfly color lightness across and within habitats. For example, within rainforests, we observed clear differences in predicted color lightness between butterfly individuals in sun versus shade microhabitats as well as large differences in lightness between crepuscular and midday hours when solar radiation was strongest. In open woodlands, as expected, we did not observe any obvious difference in predicted color lightness of butterfly individuals between sun/shade conditions or crepuscular/midday hours, which is likely due to the overall uniformity of heat and radiation exposure in these open habitats. Therefore, relative to the closed rainforest, the comparatively homogenous environment of open woodlands yields lower variability in traits such as color lightness. Across the tropics, large areas of rainforests continue to be selectively logged (Hansen, Stehman, & Potapov, 2010; Peh et al., 2011; Pert, Butler, Bruce, & Metcalfe, 2012). This not only undermines critical life‐cycle processes, but also exposes these communities with long evolutionary histories of low‐light environments to high levels of solar radiation and temperature, for which they are neither acclimated nor adapted to. Our findings suggest that deforestation and selective logging could reduce the availability of thermally buffered microhabitats for tropical species (Gardner et al., 2009; Huey et al., 2009; Scheffers, Edwards, et al., 2014; Scheffers, Evans, et al., 2014) and dramatically increase their exposure to direct solar radiation (Brown, 1993; Carlson & Groot, 1997). Both human and natural disturbances can open the canopies of rainforests, which can present physiological challenges to closed‐forest butterflies (Koh, 2007).

Morphological traits are the result of “long‐term” evolutionary influences (phylogenetic component) as well as more recent adaptations or responses to the environment (specific component) (Zeuss et al., 2014). The effect of historic climate on biological patterns in the AWT is well understood (Schneider, Williams, Bermingham, Dick, & Moritz, 2005; Williams, Bolitho, & Fox, 2003) with all analyses conducted to date showing that fluctuations in rainforest extent during the Quaternary Period has been the single largest determinant of current regional‐scale patterns of vertebrate assemblage structure and biodiversity (Graham, VanDerWal, Phillips, Moritz, & Williams, 2010; Schneider, Cunningham, & Moritz, 1998; Schneider & Moritz, 1999; Schneider et al., 2005). Importantly, the predominant effect of mid–late Pleistocene climate fluctuations was extinction rather than speciation with many communities effectively filtered by advantageous or disadvantageous traits (Moritz, Patton, Schneider, & Smith, 2000; Williams & Pearson, 1997).

We partitioned the variance in color lightness and wingspan into phylogenetic and species‐specific components in order to determine the influence of phylogenetic inertia on our observed patterns of color lightness and body size and found that both the phylogenetic and species‐specific components showed differences in traits between hot and cold time periods and spatial scales. The phylogenetic component is manifested as different species prevalence between rainforest and woodland habitats, whereas the specific components of the measured traits (especially color lightness on the ventral wing and wing size) exhibited clear and consistent patterns across the environmental gradients, suggesting that dark colors and large body sizes are favored in cool environments under the influence of recent climatic variation. The one exception to these patterns was individuals sampled in shady conditions in open woodlands during midday. These butterflies represent the darkest group, counter to the expectation that they might be more active in the morning or in closed rainforest; however, this result is limited by a small sample size dominated by a few individuals of dark‐colored butterfly species (*Hypolimnas bolina* and *Hypocysta adiante*). Color lightness and body size in butterflies are highly responsive to both historic and current climates in the AWT, which provides convincing support that human‐induced disturbances that change ambient temperature and exposure to solar radiation could filter communities by these traits (Zeuss et al., 2014). Tropical ectotherms have high projected risk of exceeding thermal limits under climate warming (Bonebrake & Deutsch, 2012; Huey et al., 2009; Tewksbury, Huey, & Deutsch, 2008), and traits that are beneficial under cool environments may lose their value or become costly as the climate warms (Willis et al., 2015). Behavioral studies suggest that butterflies need to keep their body temperatures within a range of 30–40°C for flight (Kingsolver, 1983; Kingsolver & Watt, 1983; Watt, 1968). Our thermal experiment demonstrated that the body temperature of dark butterfly species increases rapidly in a short time period. Under climate change, additional increases to already high temperatures could be consequential for some species in that warming would further reduce the available hours for critical life‐cycle processes such as foraging, mating, and reproduction (Bonebrake et al., 2014).

There are other factors that structure butterfly traits at both local and broad scales which were not considered in our study but should be acknowledged to facilitate complementary research. For example, in this study, we focused on morphology at the species and community/assemblage level rather than on variation among individuals and sexes (e.g., Ellers & Boggs, 2004). A more detailed trait analysis considering intraspecific variance in lightness and size could also provide valuable information on how plastic these traits might be in variable environments. Moreover, the color lightness in this study only captures variation in pigment coloration, yet structural or iridescence might also influence thermoregulation and fitness in butterflies (Tamáska et al., 2013). Additionally, biotic factors such as predation and competition exert selective pressures on species morphology and can shape the morphological pattern of assemblages across environmental gradients (Chai & Srygley, 1990; Cook, Mani, & Varley, 1986). This is especially true for ectotherms where mimicry and camouflage are key determinants of morphology (Pfennig, 2012; Stuart‐Fox & Moussalli, 2009; Stuart‐Fox, Whiting, & Moussalli, 2006, The Heliconius Genome Consortium 2012). The combined effects of thermoregulation and species interactions on morphology are complex (Lindstedt, Lindström, & Mappes, 2009; Stuart‐Fox & Moussalli, 2009) and should be investigated together to more fully understand trait variation of ecological communities across thermal gradients and community responses to climate change.

Our study emphasizes the important role of microclimates and habitat in shaping the color lightness and body sizes of tropical butterflies. Within a phylogenetic framework, we found bigger and darker butterflies in cool microhabitats, suggesting that these traits confer some benefit for ectotherms in cool environments at fine spatial scales, even in lowland tropical environments. These results together highlight the complex interactions between morphology and microclimate, across ecological and evolutionary time scales, and how species vulnerability to climate change could be mediated by these interactions.

## Funding Information

This work was generously supported by the Centre for Tropical Biodiversity and Climate Change at James Cook University, Earthwatch, the Australian Government's National Environment Research Program, the Australian Research Council, and the Hong Kong Research Grants Council (HKU 760213).

## Conflict of Interest

None declared.
