# Supplementary material for: Cool habitats support darker and bigger butterflies in Australian tropical forests
Source: Ecol Evol. 2016 Oct 14;6(22):8062–74. doi: 10.1002/ece3.2464 (PMC5108258; doi:10.1002/ece3.2464)
Supplement: Supplementary file 1 [file ECE3-6-8062-s001.docx]

Figure S1 Abundance of butterflies for each family across habitats (*n=*408).


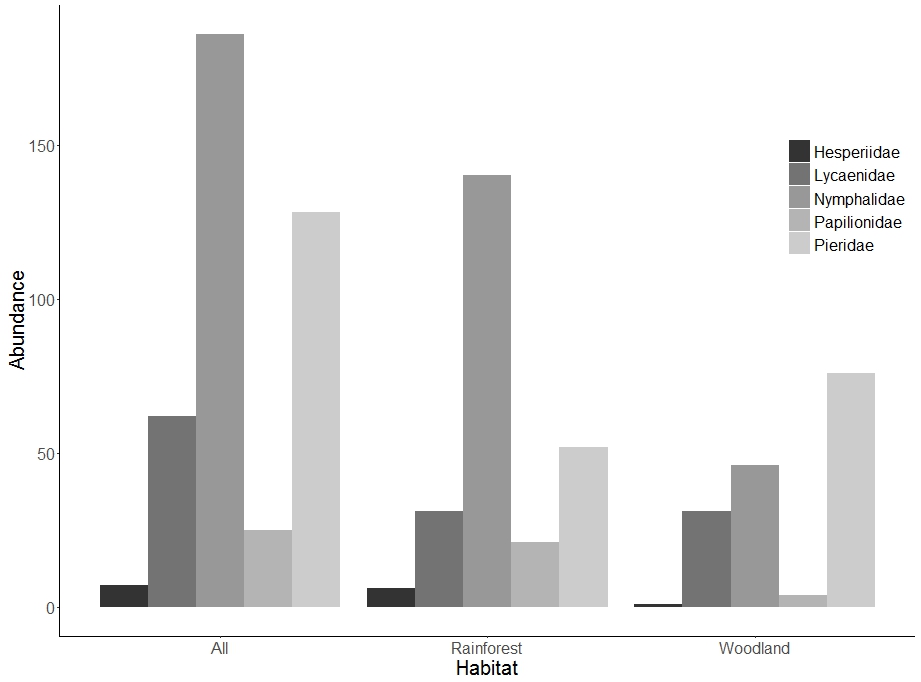


Figure S2. Species richness of each family across habitats (*n=*46).


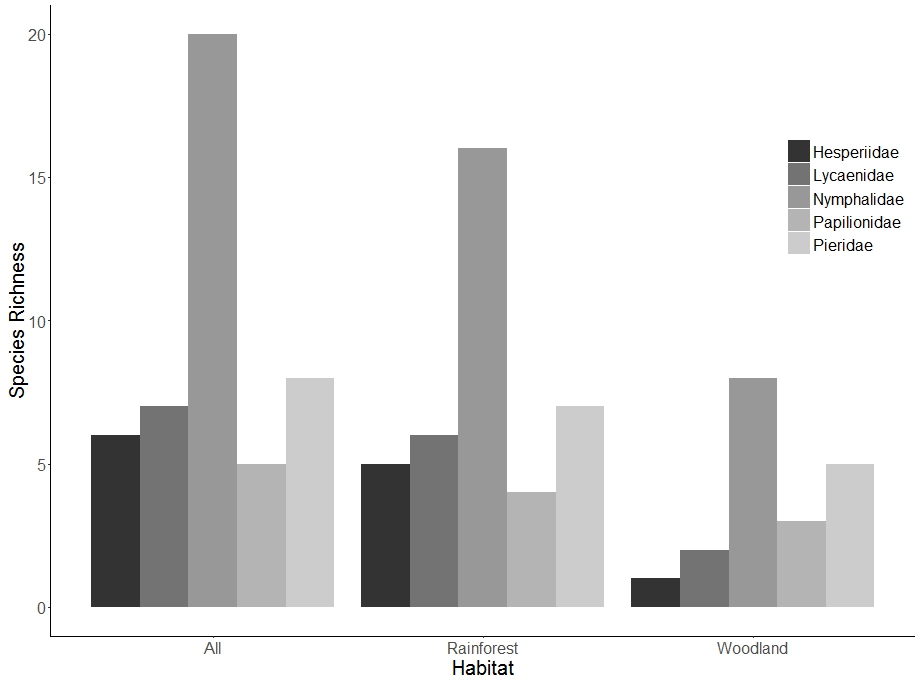


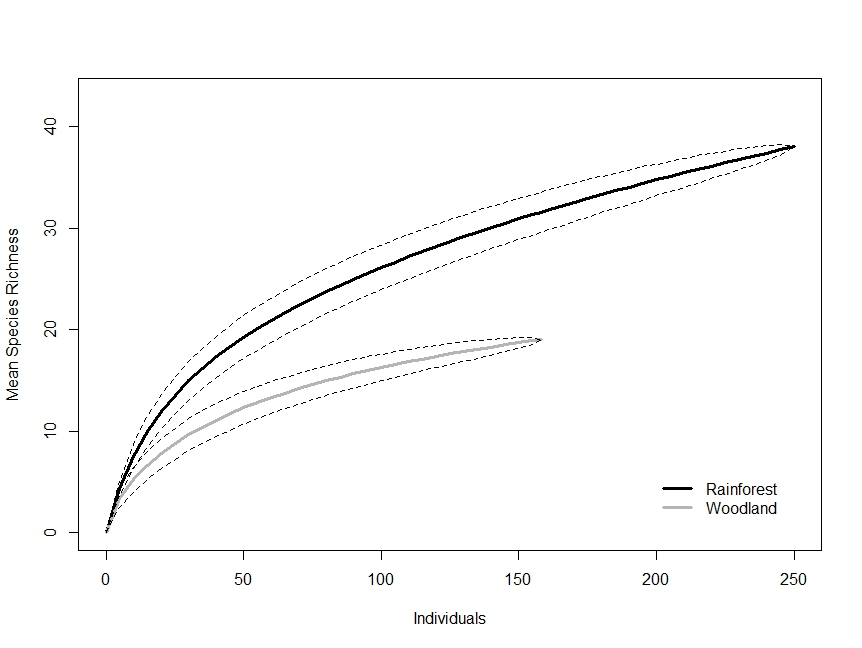
Figure S3. Rarefaction curves of butterfly species richness across habitats.

Figure S4. NMDS plot of butterfly assemblages by site for both habitats.


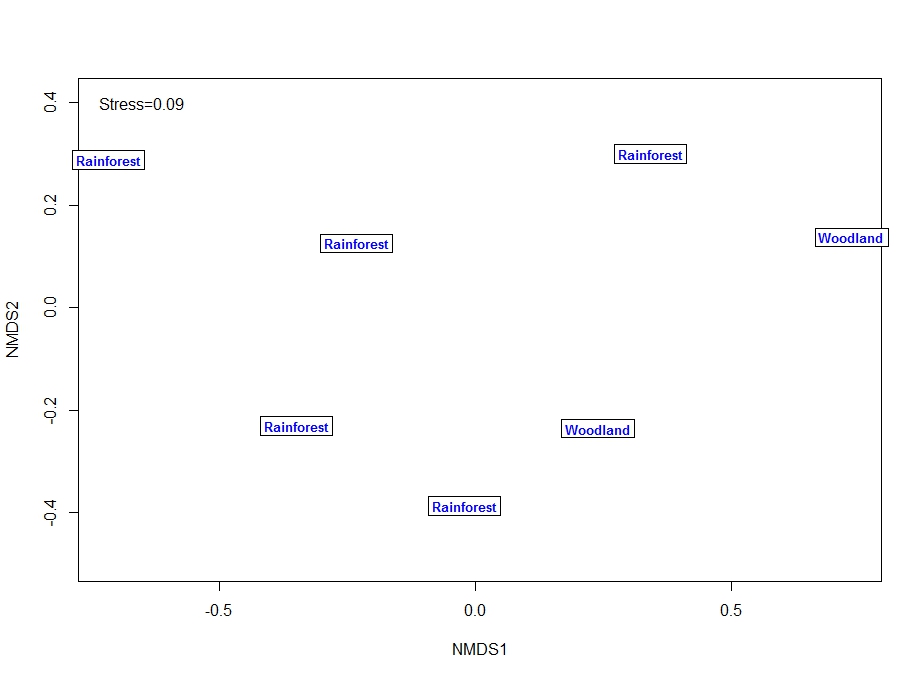


Figure S5. Bayesian 50% majority-rule consensus tree generated from nine genes (COI, COII, 16s, NADH5, CAD, EF1α, GAPDH, IDH and *wingless*). Numbers on branches indicate posterior probabilities (PP).


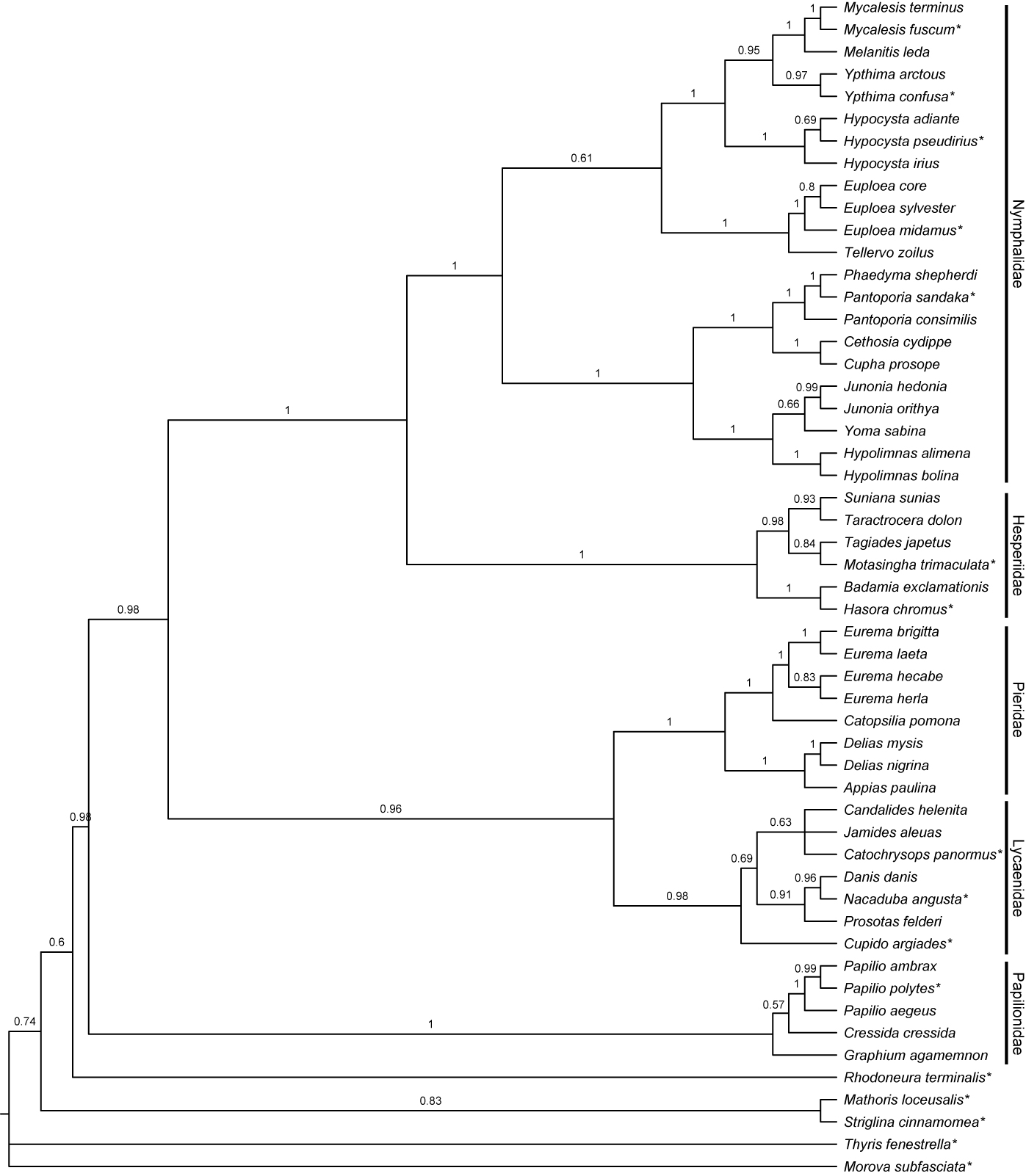


Figure S6. Predicted ambient temperatures experienced by butterflies during sampling across habitats and time (*n=*402). Pointplots indicate the mean and errobar indicate the standard error.


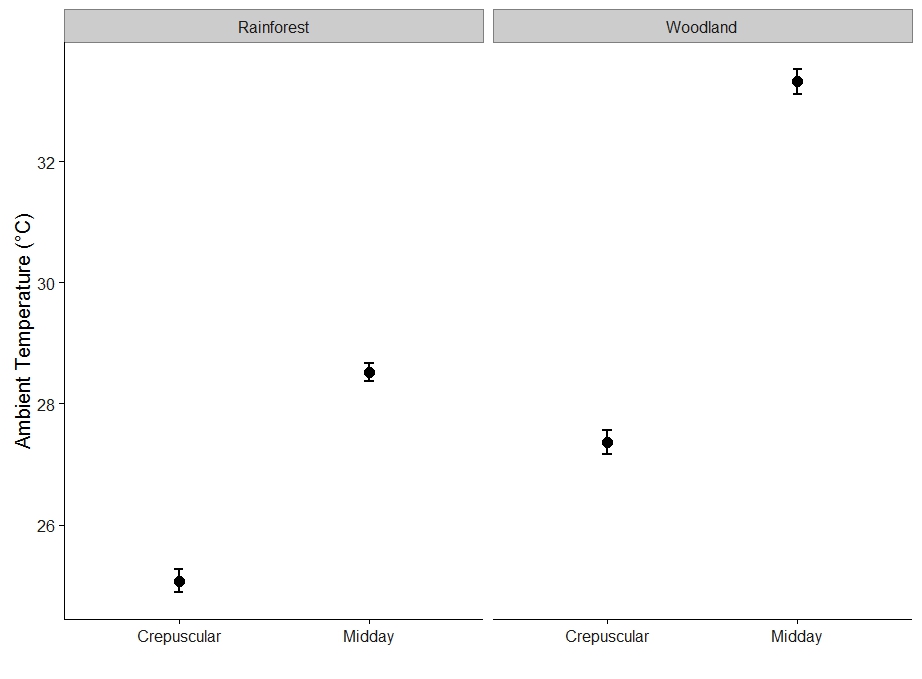


Figure S7. Predicted mean color lightness with standard error based on the best multiple linear regression model for dorsal (A) and ventral (B) sides of butterfly species across habitats and time (higher color lightness values indicate lighter colors) (*n=*100).


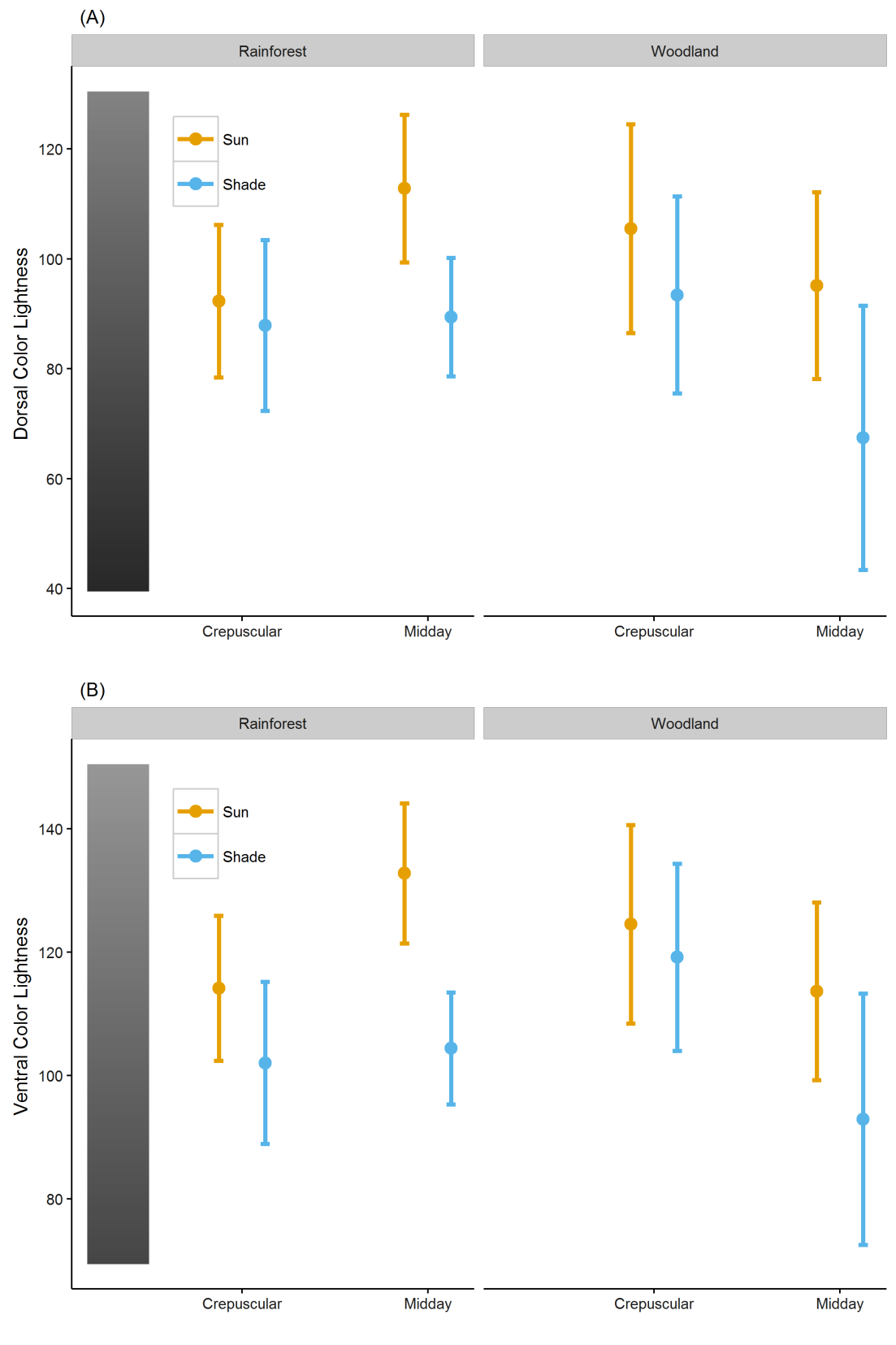


Figure S8. Predicted mean wingspan with standard error of butterfly species based on one of the best multiple linear regression models across habitats and time (n=107).


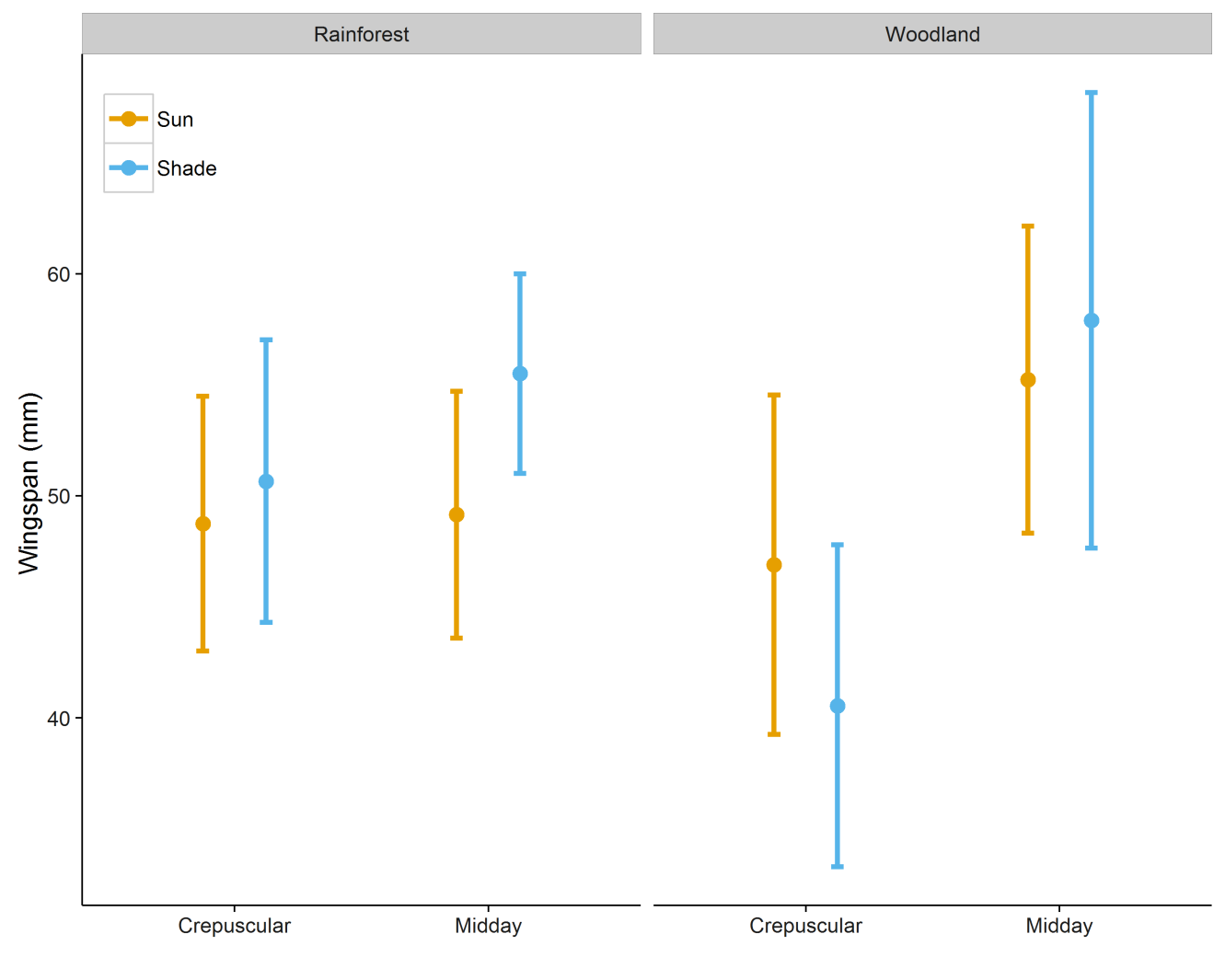


Figure S9. Phylogenetic signal (lambda) with standard error calculated based on 1000 phylogenetic trees for butterfly dorsal and ventral color lightness and wingspan.


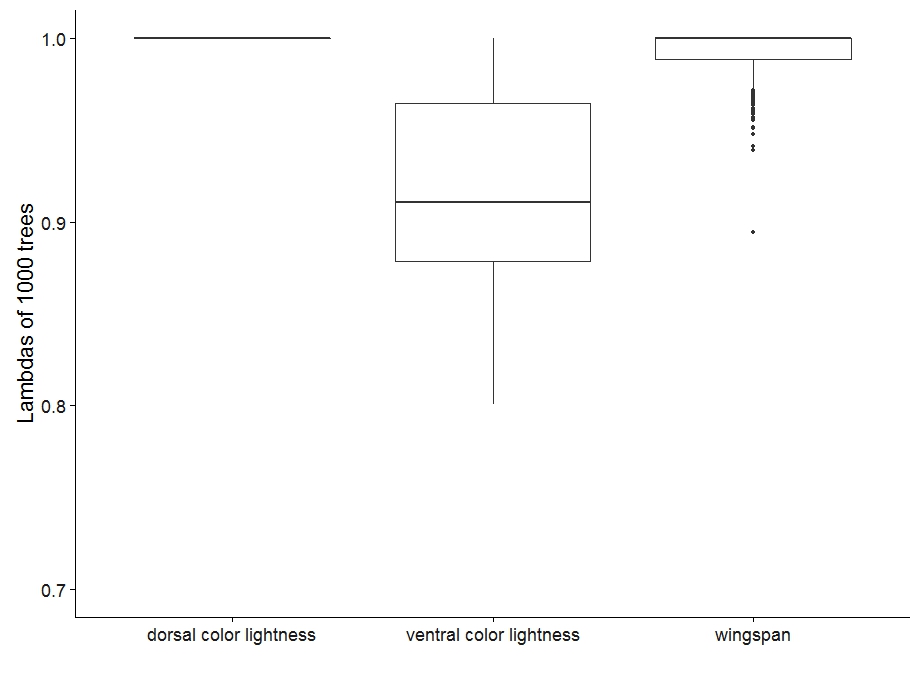


Figure S10. Raw data of species with abundance across environments.


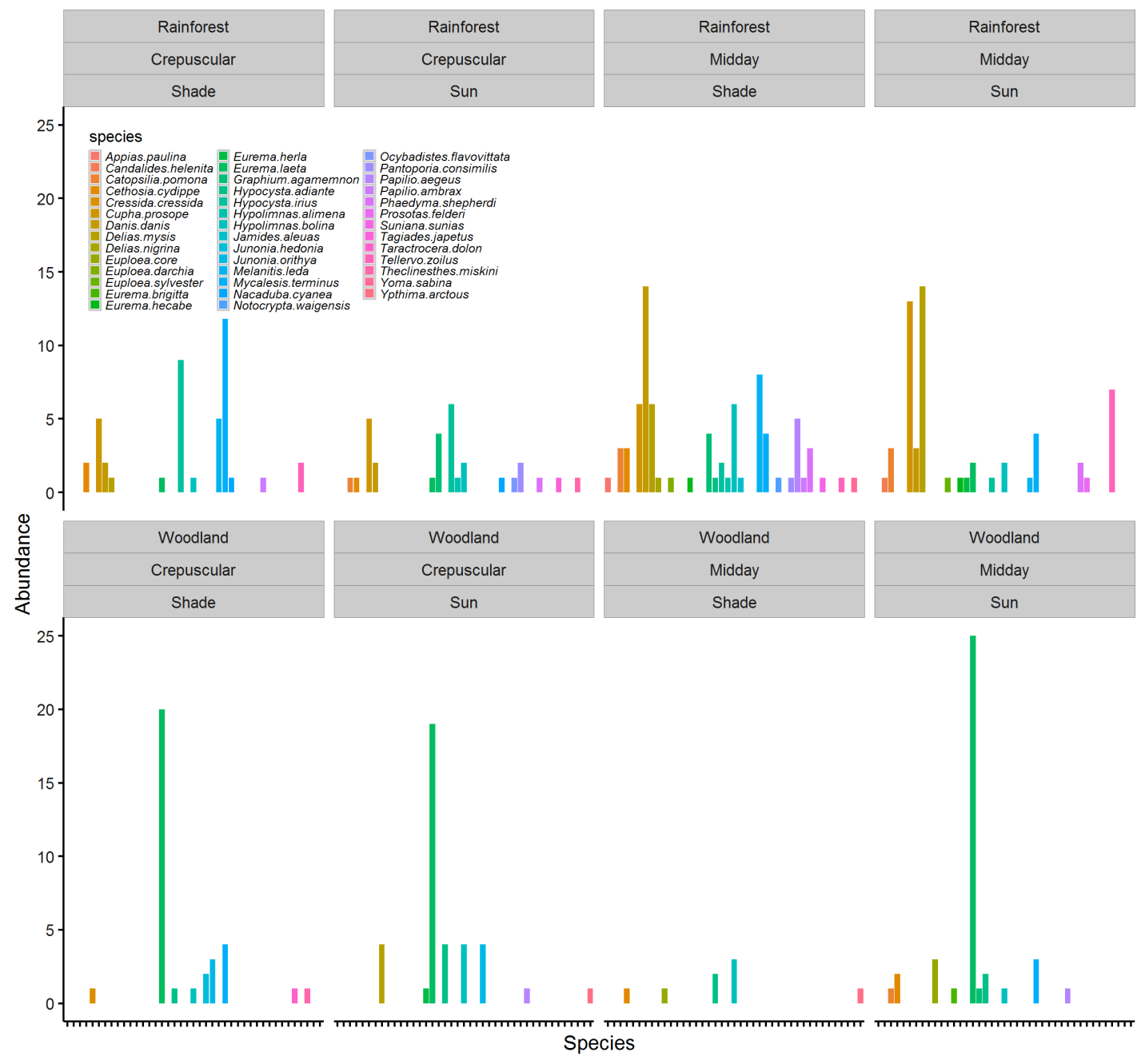


Table S1. Five best multiple linear regression models between environmental factors and butterfly wingspan.

| Factors | Number of factors | AIC | ∆AIC | Weights |
| --- | --- | --- | --- | --- |
| Habitat, Time, Haibtat*Time | 3 | 3141.1 | 0 | 0.686 |
| Habitat, Time | 2 | 3143.1 | 2 | 0.252 |
| Habitat, Time, Sun, Habitat*Time, Habitat*Sun, Time*Sun, Habitat*Time*Sun | 7 | 3147.5 | 6.4 | 0.028 |
| Habitat | 5 | 3148.3 | 7.2 | 0.019 |
| Habitat, Time, Sun, Habitat*Sun,Time*Sun | 5 | 3148.8 | 7.7 | 0.015 |

AIC, Akaike information criterion; ∆AIC, Akaike differences; Weights, Akaike weights.
